# The burden and trends of gastric cancer from 1990 to 2021 in China and globally: a cross-sectional study

**DOI:** 10.3389/fmed.2025.1533544

**Published:** 2025-05-27

**Authors:** Yulai Yin, Xiaoyu Zhang

**Affiliations:** ^1^Cangzhou Central Hospital Affiliated to Hebei Medical University, Cangzhou, China; ^2^Department of Thyroid and Breast Surgery III, Cangzhou Central Hospital, Cangzhou, China

**Keywords:** gastric cancer, disease burden, incidence, prevalence, socio-demographic index

## Abstract

**Objective:**

This study aimed to systematically analyze the trends in gastric cancer burden in China and globally from 1990 to 2021 using data from the Global Burden of Disease (GBD) database, and to forecast future trends from 2022 to 2036. The findings provide a scientific basis for developing targeted gastric cancer prevention and control policies.

**Methods:**

Age-standardized incidence rates (ASIR) and age-standardized mortality rates (ASMR) for gastric cancer from 1990 to 2021 were extracted from the GBD database. These data were complemented by GLOBOCAN 2022 statistics to analyze the spatiotemporal characteristics of gastric cancer burden across time, sex, and regions. An autoregressive integrated moving average (ARIMA) model was employed to predict trends from 2022 to 2036.

**Results:**

From 1990 to 2021, both ASIR and ASMR for gastric cancer demonstrated significant declines in China and globally. In China, the ASIR decreased from 70.458 per 100,000 in 1990 to 40.125 per 100,000 in 2021 and is projected to further decrease to 25.432 per 100,000 by 2036. Similarly, the ASMR declined from 60.781 per 100,000 in 1990 to 30.214 per 100,000 in 2021 and is forecasted to reach 18.673 per 100,000 by 2036. Globally, the ASIR fell from 35.284 per 100,000 in 1990 to 20.157 per 100,000 in 2021, with a predicted decrease to 12.493 per 100,000 by 2036. Meanwhile, the global ASMR dropped from 30.651 per 100,000 in 1990 to 15.372 per 100,000 in 2021 and is expected to reach 10.284 per 100,000 by 2036. The study also identified significant gender disparities, with males experiencing a substantially higher burden of gastric cancer than females in both China and globally. Furthermore, the age of peak incidence gradually shifted to older age groups, and high-income regions exhibited greater declines in gastric cancer burden compared to low-income regions, highlighting notable regional disparities.

**Conclusion:**

Over the past three decades, significant progress has been made in reducing the burden of gastric cancer in China and globally, with declining incidence and mortality rates. These trends are expected to continue in the coming years. However, low-and middle-income countries have seen more limited reductions, with some regions even experiencing increasing burdens. The findings underscore the need for enhanced public health policies focused on preventing *Helicobacter pylori* infections, promoting healthy dietary and lifestyle changes, and expanding the coverage of early screening programs. This study provides critical evidence to support the optimization of global and regional gastric cancer prevention and control strategies.

## Graphical abstract

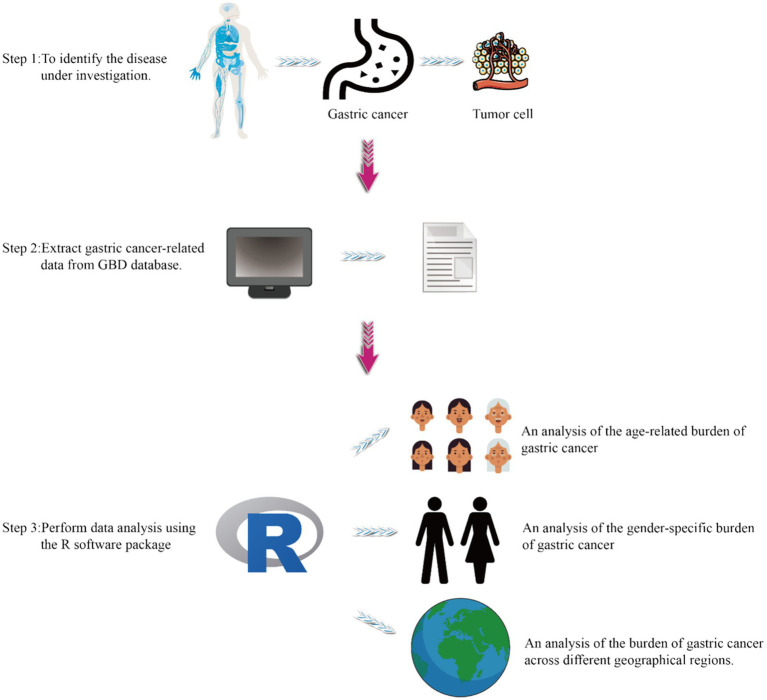

## 1 Introduction

Gastric cancer, a malignant tumor originating from the epithelial lining of the stomach mucosa, primarily encompasses adenocarcinoma, signet ring cell carcinoma, and, less commonly, undifferentiated carcinoma. Its pathogenesis is complex and closely associated with various risk factors, including *Helicobacter pylori* (HP) infection, unhealthy dietary habits (e.g., high salt intake and consumption of pickled foods), genetic predisposition, smoking, and alcohol consumption (1, 2). Gastric cancer is often asymptomatic in its early stages, lacking specific clinical signs, but once advanced, it frequently presents with symptoms such as weight loss, nausea, vomiting, and epigastric pain, significantly impairing quality of life and survival outcomes (3). Despite notable advancements in diagnostic and therapeutic approaches in recent years, the overall prognosis remains poor, particularly for patients diagnosed at intermediate or advanced stages.

According to GLOBOCAN 2022 data, gastric cancer is among the leading malignancies worldwide in terms of incidence and mortality (4). In 2020, there were 1,089,103 newly diagnosed cases globally, accounting for 5.6% of all cancer diagnoses, and 768,793 deaths, constituting 7.7% of all cancer-related fatalities. East Asia exhibits the highest incidence rates, with China, Japan, and South Korea collectively contributing approximately 75% of global cases. In China, gastric cancer is the second most prevalent malignancy following lung cancer, with an estimated 479,000 new cases and 373,000 deaths reported in 2020, representing over 40% of the global gastric cancer burden. These high incidence and mortality rates underscore the urgent need to address gastric cancer as a critical public health challenge globally, particularly in East Asia.

In recent years, advances in early screening and therapeutic strategies have improved the detection rates of early-stage gastric cancer, especially in high-incidence regions, due to the widespread application of endoscopic techniques (5, 6). However, the overall effectiveness of prevention and treatment remains limited, as most patients are diagnosed at intermediate or advanced stages, missing the optimal window for curative treatment. Furthermore, primary prevention measures for gastric cancer, such as *H. pylori* eradication and dietary modifications, have not been widely implemented in many low-and middle-income countries, posing significant challenges to global gastric cancer control efforts.

Against this backdrop, this study aims to analyze the trends in the burden of gastric cancer in China and globally from 1990 to 2021, providing a scientific basis for precise prevention and control strategies. The primary objective is to explore the temporal and spatial distribution patterns of gastric cancer burden, identifying priority areas and populations for targeted interventions. Additionally, this study incorporates projections of disease burden trends to offer actionable recommendations for policymakers, thereby optimizing global efforts in gastric cancer prevention and control. By promoting resource allocation and improving survival outcomes among high-risk populations, this research carries significant implications for public health.

## 2 Materials and methods

### 2.1 Data sources

The Global Burden of Disease (GBD) study, led by the Institute for Health Metrics and Evaluation (IHME), provides comprehensive and comparable global health metrics. The data for this study were obtained from the GBD 2021 dataset (https://ghdx.healthdata.org/gbd-2021), which offers critical insights into mortality, disease burden, and risk factors across different regions and populations (7). This extensive dataset includes age-and sex-specific data on the incidence, prevalence, and mortality of 369 diseases and injuries across 204 countries and territories.

For this analysis, data on age-standardized incidence, prevalence, mortality, and disability-adjusted life years (DALYs) for gastric cancer were extracted from the GBD 2021 dataset, spanning 1990–2021 for both China and the global population. The analysis included all age groups and sexes to comprehensively assess trends and provide actionable insights.

### 2.2 Statistical analysis

This study extracted data from the GBD database on the incidence, prevalence, mortality, and disability-adjusted life years (DALYs) of gastric cancer in China and globally. The analysis included corresponding age-standardized metrics, such as the age-standardized incidence rate (ASIR), age-standardized prevalence rate (ASPR), age-standardized mortality rate (ASMR), and age-standardized disability-adjusted life year rate (ASDR), as well as crude metrics by age group, including crude incidence rate (CIR), crude prevalence rate (CPR), crude mortality rate (CMR), and crude disability-adjusted life year rate (CDR).

To evaluate disease burden trends, the Joinpoint Regression Program (National Cancer Institute, Rockville, MD, United States) was used to calculate the average annual percentage change (AAPC) and its corresponding 95% confidence intervals (95% CIs). Log-transformed age-standardized indicators were fitted to a regression model of the form ln(*y*) = *α* + *βx* + *ε*, where *y* represents the respective age-standardized indicator, *x* represents the calendar year, and *ε* represents the error term. AAPC was calculated as 100 × (exp(*β*) − 1), with 95% CIs derived from the model. An AAPC with a 95% CI >0 indicates an increasing trend, <0 indicates a decreasing trend, and a 95% CI including 0 suggests a stable trend.

For forecasting future trends, an auto-regressive integrated moving average (ARIMA) model was employed (8). The ARIMA model is widely used for time series analysis and is particularly suitable for predicting future trends based on historical data. The model parameters were determined through autocorrelation and partial autocorrelation function analysis, with parameter optimization based on the Akaike information criterion (AIC) and Bayesian information criterion (BIC). After training, the ARIMA model was applied to predict the incidence, mortality, and DALYs of gastric cancer over the next 15 years, providing quantitative projections of future disease burden.

All statistical analyses and visualizations were performed using R statistical software (version 4.3.2) and the Joinpoint Regression Program (version 4.9.1.0) (9–11). A *p*-value <0.05 was considered statistically significant.

### 2.3 Data disclosure statement

The data used in this study were derived from the GBD 2021 dataset (https://ghdx.healthdata.org/gbd-2021), which does not include identifiable personal information. The original studies associated with this dataset have been approved by relevant ethics committees. Therefore, this study did not require additional ethical approval.

## 3 Results

### 3.1 Description of gastric cancer burden in China and globally

From 1990 to 2021, the burden of gastric cancer exhibited a declining trend in both China and globally, with China achieving more pronounced progress in reducing incidence, mortality, and disability-adjusted life years (DALYs). In terms of incidence, the number of cases in China increased from 407,471 to 611,799, while globally, cases rose from 980,899 to 1,230,233. Despite this rise in absolute case numbers, the age-standardized incidence rate (ASIR) in China dropped significantly from 48.026 to 29.053 (AAPC: −1.6%), compared to a global decline from 24.763 to 14.328 (AAPC: −1.8%). Regarding prevalence, the total number of cases in China doubled to 1,226,056, while global prevalence increased to 2,393,213. The age-standardized prevalence rate (ASPR) declined to 57.225 in China (AAPC: −0.5%) and to 27.581 globally (AAPC: −1.3%). In terms of mortality, deaths in China rose from 374,066 to 445,013, and globally, from 854,185 to 954,374. However, the age-standardized mortality rate (ASMR) in China showed a more pronounced decrease, dropping from 46.048 to 21.509 (AAPC: −2.4%), compared to a global reduction from 22.006 to 11.199 (AAPC: −2.2%). For DALYs, the total in China slightly decreased from 10,773,457 to 10,642,127, while globally, DALYs dropped from 23,237,292 to 22,786,633. The age-standardized DALY rate (ASDR) saw a greater reduction in China (AAPC: −2.8%) compared to the global rate (AAPC: −2.4%). These trends reflect China’s significant advancements in managing the burden of gastric cancer over the past three decades (see Table 1).

**Table 1:** All-age case numbers, age-standardized incidence, prevalence, mortality, and DALY rates, and corresponding AAPC for gastric cancer in China and globally in 1990 and 2021.

Location	Measure	1990		2021		1990–2021 AAPC
		All-ages cases	Age-standardized rates per 100,000 people	All-ages cases	Age-standardized rates per 100,000 people	
		*n* (95% CI)	*n* (95% CI)	*n* (95% CI)	*n* (95% CI)	*n* (95% CI)
China	Incidence	407,471 (337,565–477,569)	48.026 (40.215–56.685)	611,799 (471,966–765,562)	29.053 (22.423–36.2)	−1.6 (−1.7 to −1.5)
	Prevalence	615,217 (503,482–720,422)	67.17 (55.347–78.413)	1,226,056 (943,897–1,546,818)	57.225 (44.183–71.988)	−0.5 (−0.7 to −0.3)
	Deaths	374,066 (310,921–442,251)	46.048 (38.88–54.434)	445,013 (344,736–555,834)	21.509 (16.663–26.611)	−2.4 (−2.6 to −2.3)
	DALYS	10,773,457 (8,850,977–12,638,919)	1181.613 (978.381–1390.895)	10,642,127 (8,222,106–13,383,779)	501.26 (387.291–627.976)	−2.8 (−2.9 to −2.6)
Global	Incidence	980,899 (891,307–1,072,236)	24.763 (22.58–27.002)	1,230,233 (1,052,350–1,409,970)	14.328 (12.226–16.408)	−1.8 (−1.9 to −1.6)
	Prevalence	1,671,262 (1,524,321–1,796,161)	40.638 (37.254–43.677)	2,393,213 (2,059,698–2,771,476)	27.581 (23.75–31.887)	−1.3 (−1.4 to −1.1)
	Deaths	854,185 (772,885–939,973)	22.006 (20.028–24.187)	954,374 (821,751–1,089,577)	11.199 (9.618–12.734)	−2.2 (−2.3 to −2)
	DALYS	23,237,292 (2,060,5349–25,526,194)	559.721 (499.087–615.772)	22,786,633 (19,576,344–26,118,869)	262.748 (226.079–301.024)	−2.4 (−2.5 to −2.3)

### 3.2 Regression analysis of gastric cancer burden in China and globally using joinpoint

**Figure 1:**
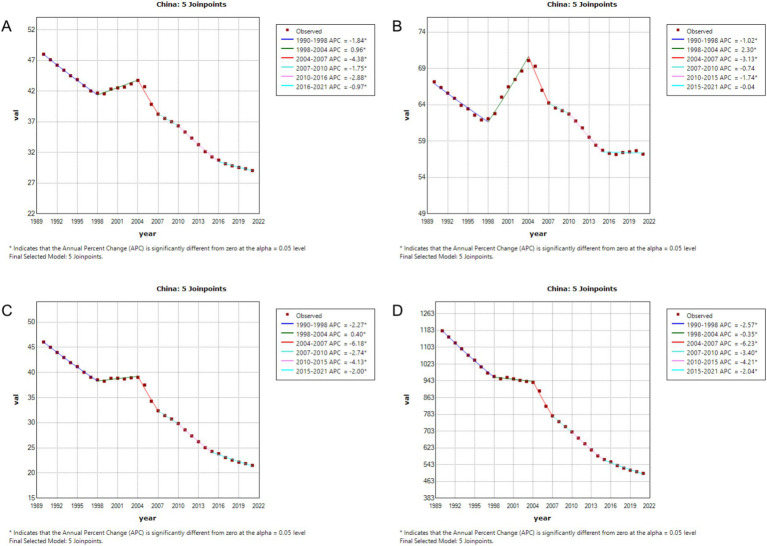
Age-standardized incidence rate (ASIR), prevalence rate (ASPR), mortality rate (ASMR), and disability-adjusted life year rate (ASDR) APCs for gastric cancer in China, 1990–2021 (* indicates *p* < 0.05, denoting statistical significance). **(A)** ASIR; **(B)** ASPR; **(C)** ASMR; **(D)** ASDR.

**Figure 2:**
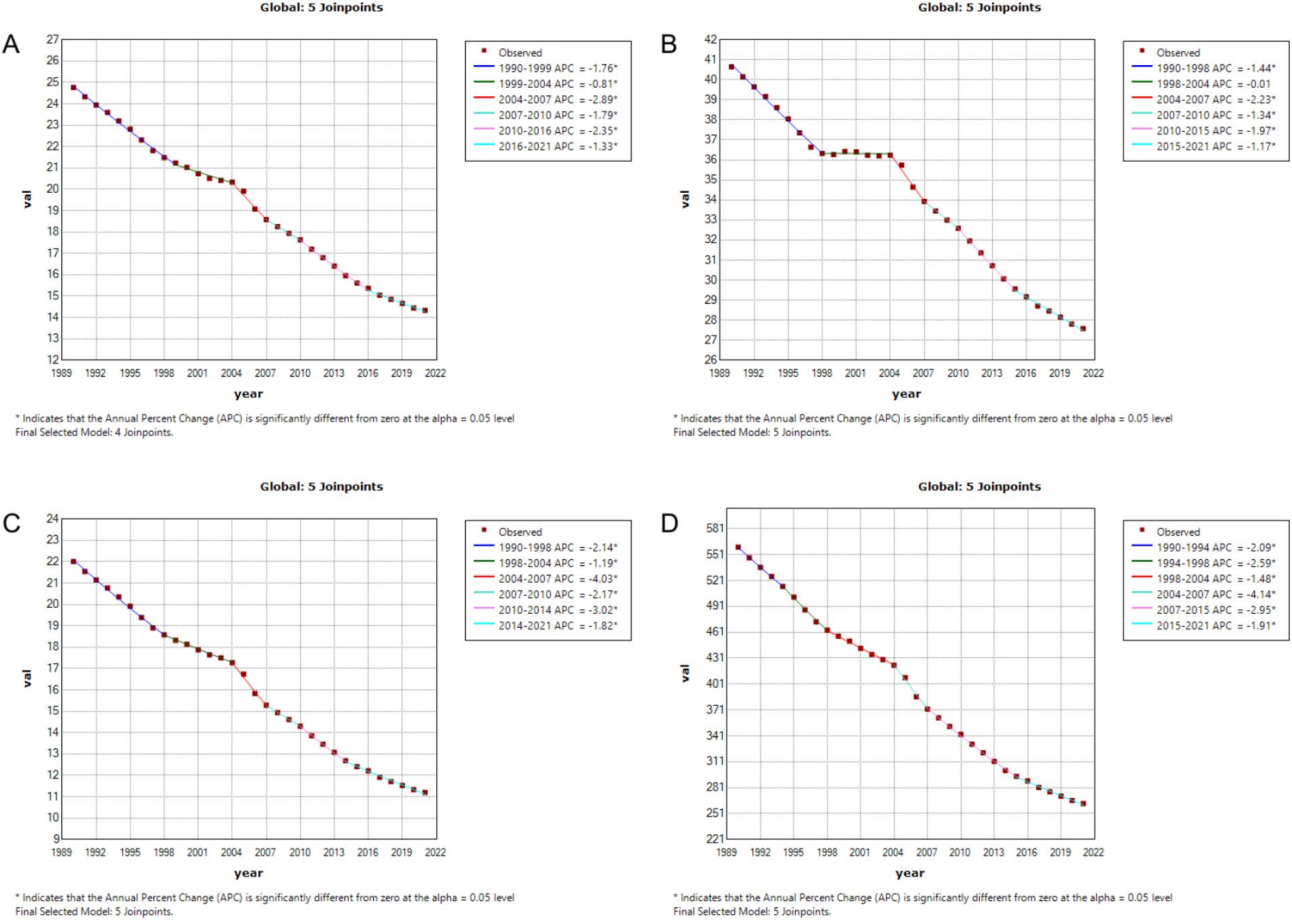
Age-standardized incidence rate (ASIR), prevalence rate (ASPR), mortality rate (ASMR), and disability-adjusted life year rate (ASDR) APCs for global gastric cancer, 1990–2021 (* indicates *p* < 0.05, denoting statistical significance). **(A)** ASIR; **(B)** ASPR; **(C)** ASMR; **(D)** ASDR.

From 1990 to 2021, the burden of gastric cancer showed significant declines across all indicators in both China and globally, with notable differences in the magnitude and characteristics of changes during specific periods. For the age-standardized incidence rate (ASIR), China’s rate decreased from 48.026 per 100,000 population (95% CI: 40.215–56.685) to 29.053 per 100,000 (95% CI: 22.423–36.2), while the global rate declined from 24.763 per 100,000 (95% CI: 22.58–27.002) to 14.328 per 100,000 (95% CI: 12.226–16.408). Despite a higher baseline incidence in China, the decline was more pronounced, especially during 2004–2007, when the annual percentage change (APC) in China was −4.38%, compared to −2.89% globally. For the age-standardized prevalence rate (ASPR), China’s rate fell from 67.17 per 100,000 (95% CI: 55.347–78.413) to 57.225 per 100,000 (95% CI: 44.183–71.988), while the global rate declined from 40.638 per 100,000 (95% CI: 37.254–43.677) to 27.581 per 100,000 (95% CI: 23.75–31.887). Although the global decline was slightly steeper, China’s absolute burden remained higher. For the age-standardized mortality rate (ASMR), China achieved a substantial reduction from 46.048 per 100,000 (95% CI: 38.88–54.434) to 21.509 per 100,000 (95% CI: 16.663–26.611), whereas the global rate dropped from 22.006 per 100,000 (95% CI: 20.028–24.187) to 11.199 per 100,000 (95% CI: 9.618–12.734). During 2004–2007, China’s APC reached −6.18%, significantly faster than the global APC of −4.03%, reflecting China’s remarkable progress in reducing gastric cancer mortality. Similarly, for the age-standardized DALY rate, China’s rate decreased from 1181.613 per 100,000 (95% CI: 978.381–1390.895) to 501.26 per 100,000 (95% CI: 387.291–627.976), while the global rate declined from 559.721 per 100,000 (95% CI: 499.087–615.772) to 262.748 per 100,000 (95% CI: 226.079–301.024). Notably, during 2004–2007, China’s APC for DALYs was −6.23%, significantly exceeding the global APC of −4.03%, underscoring the significant strides China made in reducing the burden of gastric cancer during this period, as illustrated in Figures 1, 2.

### 3.3 Trends in gastric cancer burden in China and globally

**Figure 3:**
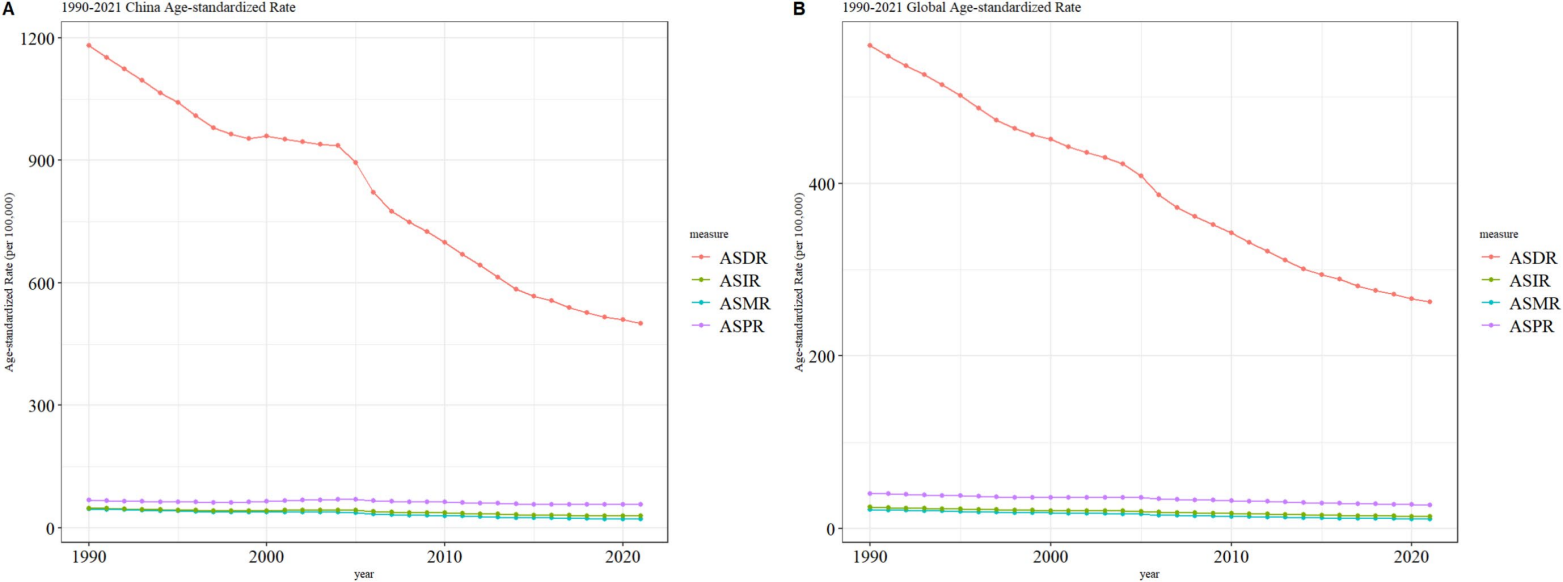
Comparative trends of age-standardized incidence rate (ASIR), prevalence rate (ASPR), mortality rate (ASMR), and disability-adjusted life year rate (ASDR) for gastric cancer in China and globally, 1990–2021.

As shown in Figure 3, the age-standardized burden of gastric cancer across all indicators exhibited significant declining trends in both China and globally from 1990 to 2021. The left panel of the figure represents trends in China, while the right panel represents global trends. The four primary indicators include the age-standardized disability-adjusted life year rate (ASDR), incidence rate (ASIR), mortality rate (ASMR), and prevalence rate (ASPR). In China, the ASDR decreased significantly from approximately 1,181 per 100,000 population in 1990 to about 501 per 100,000 in 2021, whereas the global ASDR dropped from approximately 560 per 100,000 to about 263 per 100,000 during the same period. Despite China’s higher baseline ASDR, the larger reduction highlights the country’s notable achievements in reducing health life years lost due to gastric cancer. For ASIR, China’s rate declined from approximately 48 per 100,000 to about 29 per 100,000, while the global rate fell from about 25 per 100,000 to around 14 per 100,000. Although China’s baseline incidence was higher, the greater reduction reflects the effectiveness of enhanced early screening and intervention strategies. The ASMR also showed a similar pattern, with China’s mortality rate dropping from approximately 46 per 100,000 to about 21 per 100,000, compared to a global decline from around 22 per 100,000 to about 11 per 100,000. China’s more substantial decline demonstrates improvements in medical care and the effectiveness of early treatment. In contrast, ASPR exhibited a slower reduction, with China’s prevalence rate decreasing from about 67 per 100,000 to approximately 57 per 100,000, and the global rate dropping from about 41 per 100,000 to roughly 28 per 100,000. This may reflect improvements in chronic disease management and long-term care for patients. Overall, while China’s baseline burden was significantly higher than the global average, its rate of reduction across all indicators outpaced global trends, particularly in ASDR and ASMR, showcasing notable public health progress. Globally, the decline was more gradual, especially in ASPR and ASMR, reflecting the long-term effects of sustained interventions. These similar trends underscore the shared advancements in gastric cancer prevention and control strategies worldwide. However, they also highlight the need for China to focus on precise management of high-risk populations to consolidate its gains, while global efforts should prioritize strengthening prevention strategies in low-income regions to achieve a comprehensive reduction in gastric cancer burden.

### 3.4 Gastric cancer burden by age groups in China and globally in 1990 and 2021

**Figure 4:**
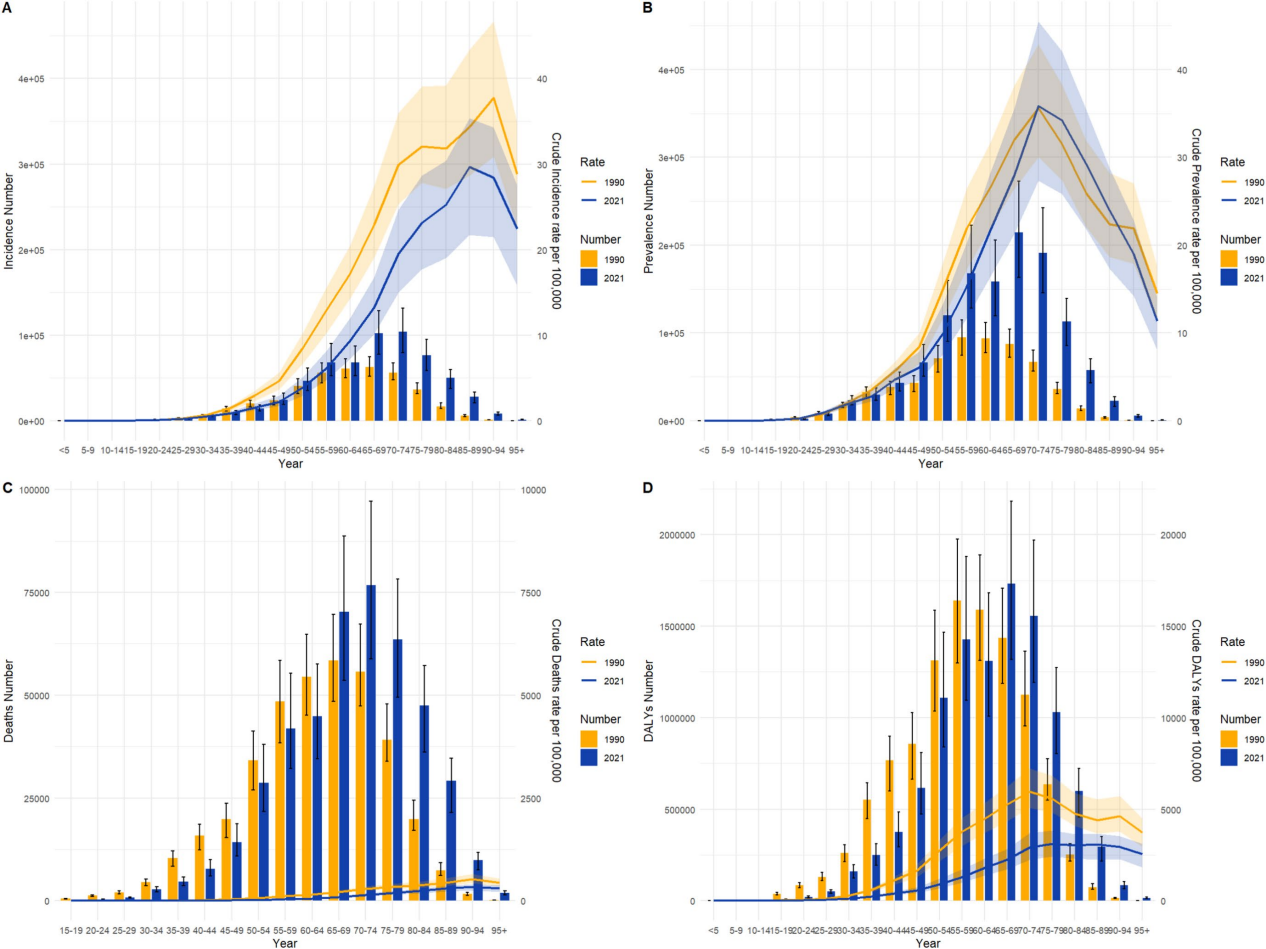
Comparison of age-specific incidence, prevalence, mortality, and DALYs counts and crude rates for gastric cancer in China by age group in 1990 and 2021.

**Figure 5:**
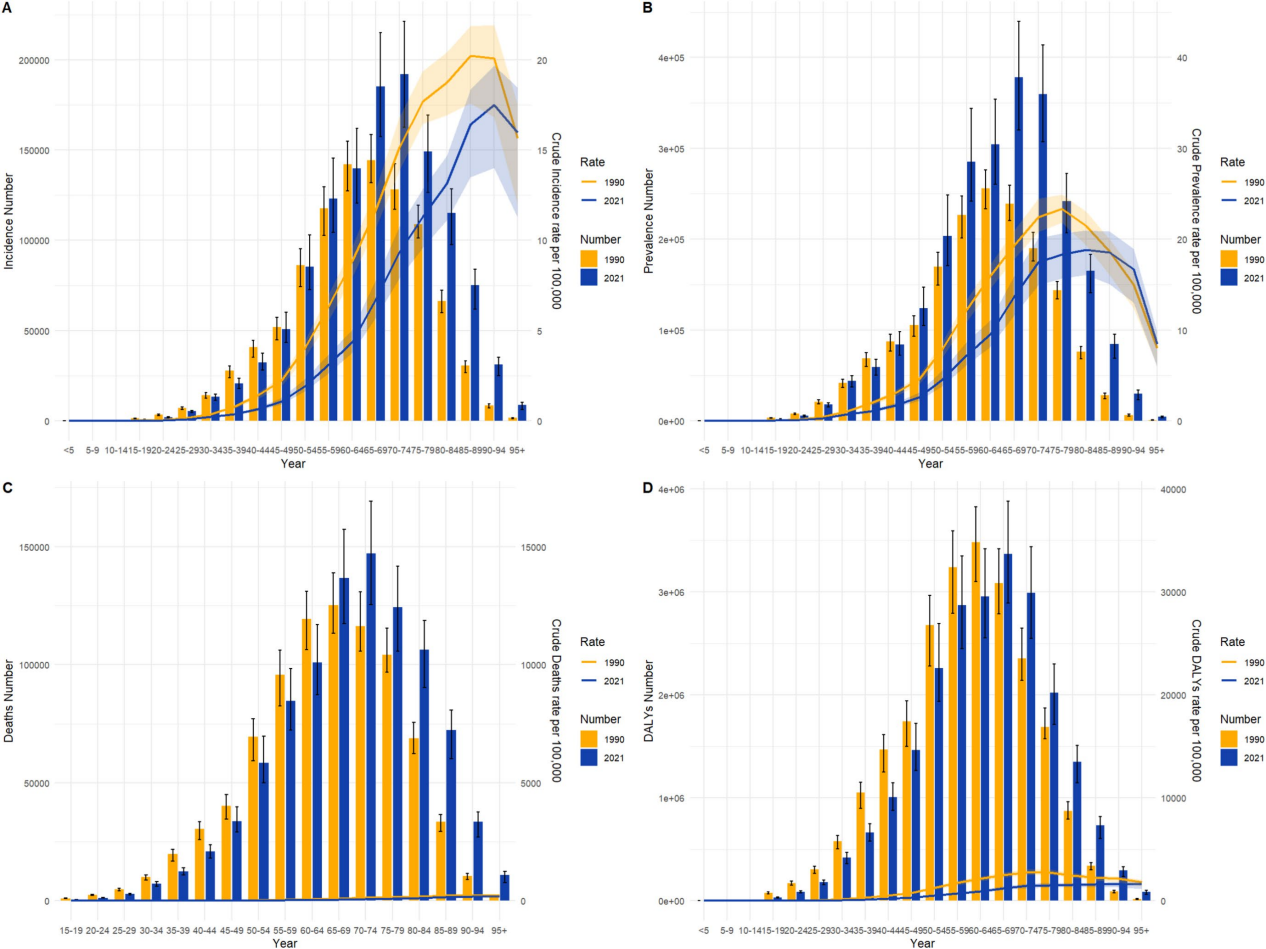
Comparison of age-specific incidence, prevalence, mortality, and DALYs counts and crude rates for gastric cancer globally by age group in 1990 and 2021.

As shown in Figures 4, 5, the age distribution of gastric cancer incidence, prevalence, mortality, and disability-adjusted life years (DALYs) underwent significant changes from 1990 to 2021, reflecting regional differences and common trends in population structure and disease prevention efforts. In terms of incidence and incidence rates, the peak age group in China shifted from 55–59 years in 1990 to 65–69 years in 2021, while globally, it shifted from 50–54 years to 60–64 years. Although incidence rates declined significantly in both China and globally, China exhibited a higher peak age group and baseline incidence rates, suggesting that its gastric cancer burden is more influenced by population aging. For prevalence and prevalence rates, China showed a markedly higher absolute prevalence than the global average, with the peak age group in 2021 occurring at 65–69 years, compared to 60–64 years globally. While the decline in prevalence rates was slightly slower in China, the delayed peak age group further underscores the impact of aging on disease management. The changes in mortality and mortality rates highlighted more pronounced regional differences and progress. In China, the absolute number of deaths increased in 2021 compared to 1990, but mortality rates declined significantly, with the peak age group shifting from 70–74 years to 75–79 years. Globally, the number of deaths and mortality rates also decreased, albeit to a lesser extent, with the peak age group moving from 65–69 years to 70–74 years. The greater decline in mortality rates in China likely reflects improvements in healthcare, expanded early screening coverage, and enhanced treatment efficacy. For DALYs and DALY rates, both China and the global population exhibited significant decreases, with the peak age group shifting from 70–74 years and 65–69 years in 1990 to 75–79 years and 70–74 years in 2021, respectively. Although China’s baseline DALY rate was substantially higher than the global rate, the reduction was more pronounced, particularly among older age groups, indicating effective mitigation of disease burden. Overall, the burden of gastric cancer in both China and globally showed a downward trend over time, with peak age groups gradually shifting to older populations. This reflects progress in global gastric cancer prevention and control measures, as well as the influence of population aging on disease burden. While China’s absolute burden remains higher than the global average, its rate of reduction is more significant, particularly in mortality and DALY rates, demonstrating substantial advancements in gastric cancer management. However, the global trend appears more stable, particularly in the long-term control of incidence and prevalence rates, highlighting sustained intervention efforts worldwide.

### 3.5 Gastric cancer burden by gender in China and globally in 1990 and 2021

**Figure 6:**
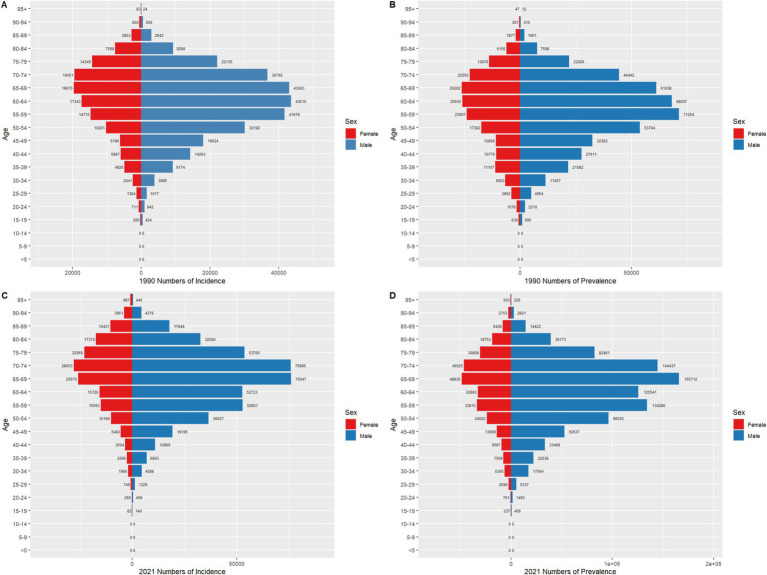
Comparison of age-and gender-specific incidence and prevalence counts for gastric cancer in China in 1990 and 2021.

**Figure 7:**
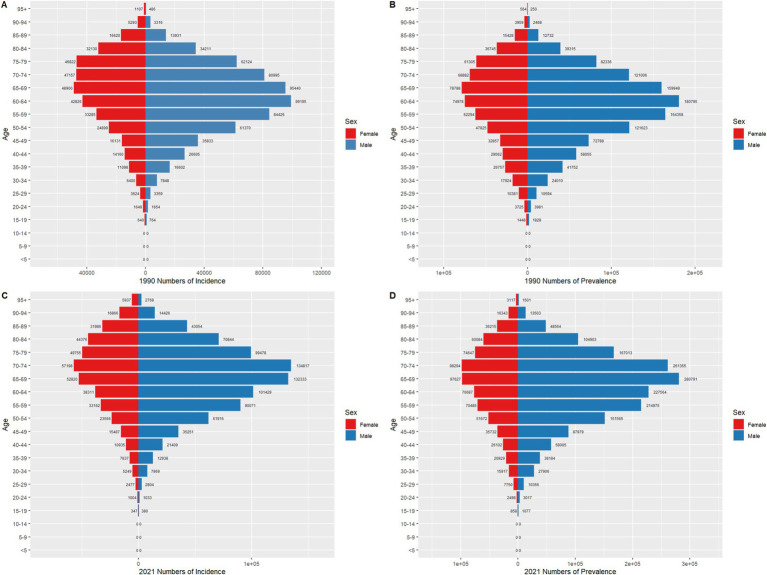
Comparison of age-and gender-specific incidence and prevalence counts for gastric cancer globally in 1990 and 2021.

From 1990 to 2021, the gender and age distribution of gastric cancer incidence and prevalence in both China and globally underwent significant changes, reflecting the combined effects of population aging and prevention measures. In terms of incidence, the peak age group for males in China shifted from 55–59 years in 1990 (43,053 cases) to 65–69 years in 2021, with cases increasing to 76,047. For females, the peak age group shifted from 50–54 years in 1990 (17,742 cases) to 65–69 years in 2021, with cases rising to 25,970. This indicates a marked delay in the age of peak incidence and an increasing gender disparity. Similarly, globally, the peak incidence age group for males shifted from 50–54 years in 1990 (99,155 cases) to 60–64 years in 2021, with cases rising to 132,333. For females, the peak shifted from 50–54 years (47,222 cases) to 60–64 years in 2021, with cases increasing to 52,020. Compared to global trends, the delay in peak age group was more pronounced in China, reflecting the deeper impact of aging on gastric cancer burden in China. For prevalence, the peak age group for males in China moved from 55–59 years in 1990 (71,543 cases) to 65–69 years in 2021, with cases reaching 125,441. For females, the peak shifted from 50–54 years in 1990 (28,207 cases) to 65–69 years in 2021, with cases rising to 38,109. Globally, the male prevalence peak age group shifted from 50–54 years in 1990 (160,895 cases) to 60–64 years in 2021, with cases increasing to 280,191. For females, it shifted from 50–54 years in 1990 (72,788 cases) to 60–64 years in 2021, with cases reaching 97,427. Both China and the global population showed significant growth in prevalence numbers over time, with a concentration in older age groups. Although the global absolute prevalence was higher than in China, the gender disparity was more pronounced in China. Overall, both China and the global population exhibited similar trends of delayed peak age and higher burden among males compared to females. However, the gender disparity was more prominent in China, and the shift in peak age groups was greater, reflecting the impact of China’s aging population and higher exposure rates to male-specific risk factors, such as smoking, alcohol consumption, and dietary habits. Globally, the absolute disease burden was larger, but gender differences were less pronounced, suggesting a more balanced effectiveness of gastric cancer prevention and control strategies. These trends are illustrated in Figures 6, 7.

### 3.6 Changes in global gastric cancer prevalence rates from 1990 to 2021

**Figure 8:**
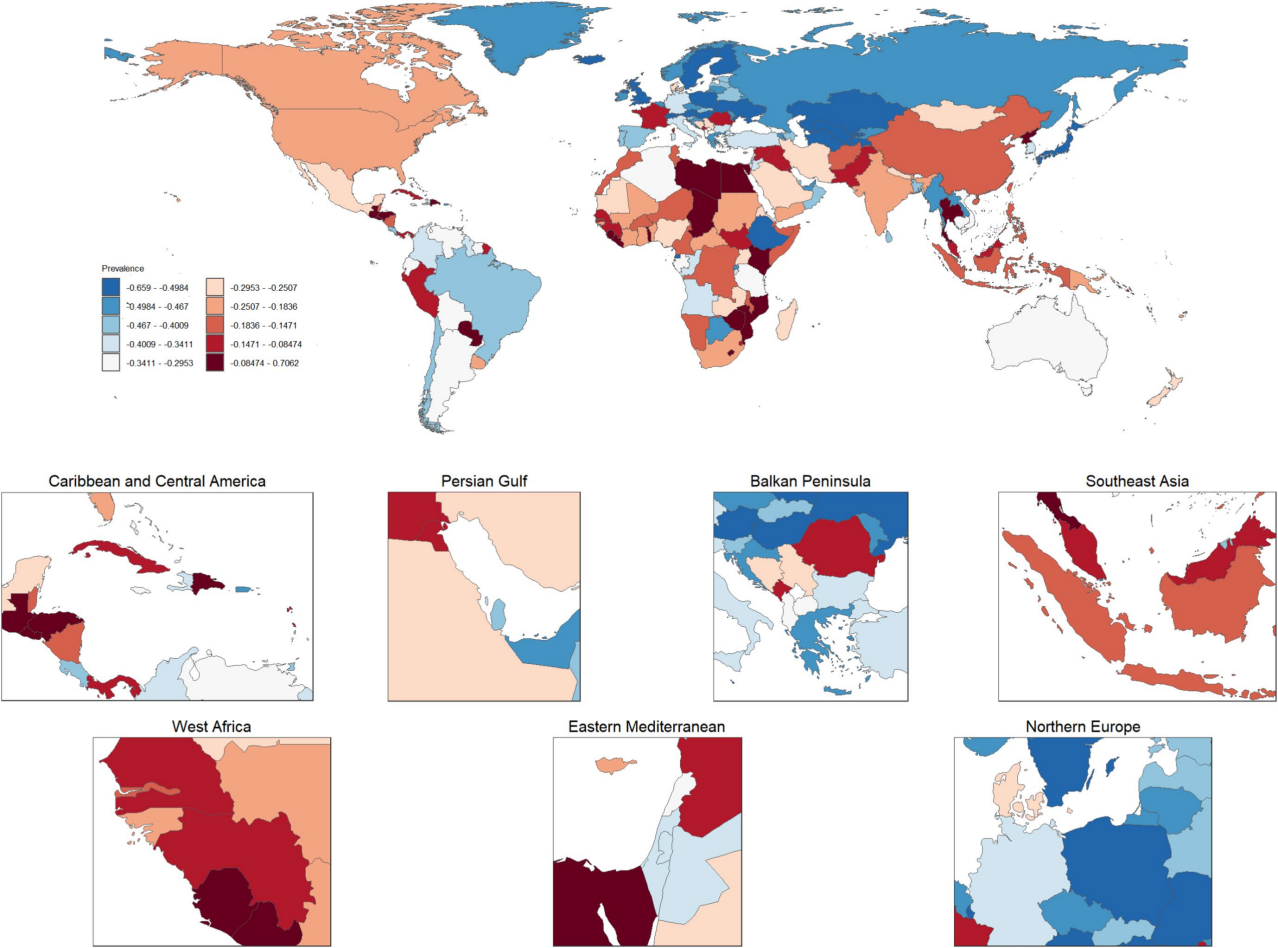
Geographic distribution of average percentage change in gastric cancer prevalence by region, 1990–2021. (This figure displays the average percentage change in gastric cancer prevalence across global regions from 1990 to 2021. Colors range from light blue to deep red, indicating varying degrees of prevalence change, with light blue representing decreases and deep red signifying significant increases. Insets further detail prevalence changes in key regions, including the Caribbean and Central America, the Persian Gulf, the Eastern Mediterranean, Southeast Asia, and Northern Europe).

As illustrated in Figure 8, the annual average percent change (AAPC) in global gastric cancer prevalence rates from 1990 to 2021 exhibited significant regional disparities. In developed regions such as North America, Northern Europe, and Western Europe, prevalence rates generally showed a declining trend. Notably, Northern European countries, including Finland, Norway, and Sweden, demonstrated significantly negative AAPC values, reflecting substantial reductions in gastric cancer burden. Similarly, countries in the Balkan Peninsula, such as Serbia and Bulgaria, also displayed declining prevalence rates consistent with trends observed across other European regions. In contrast, some developing countries and regions experienced increasing AAPC values, indicating a rising trend in prevalence rates. These included countries in Southeast Asia, such as Vietnam and Indonesia; the Middle East, such as Qatar and the United Arab Emirates; and several nations in sub-Saharan Africa. This upward trend suggests growing gastric cancer burdens in these areas. Additionally, the Caribbean and Central America exhibited mixed trends: for instance, the Dominican Republic showed a positive AAPC, whereas Costa Rica demonstrated a negative AAPC, highlighting significant variations between neighboring countries. Overall, the AAPC in global gastric cancer prevalence rates revealed distinct geographical patterns. Declines were predominant in developed regions, whereas increases were observed in some low-and middle-income countries. These trends underscore the need for tailored prevention and control strategies that address the specific needs and challenges of regions experiencing rising gastric cancer burdens.

### 3.7 Relationship between socio-demographic index and gastric cancer burden

**Figure 9:**
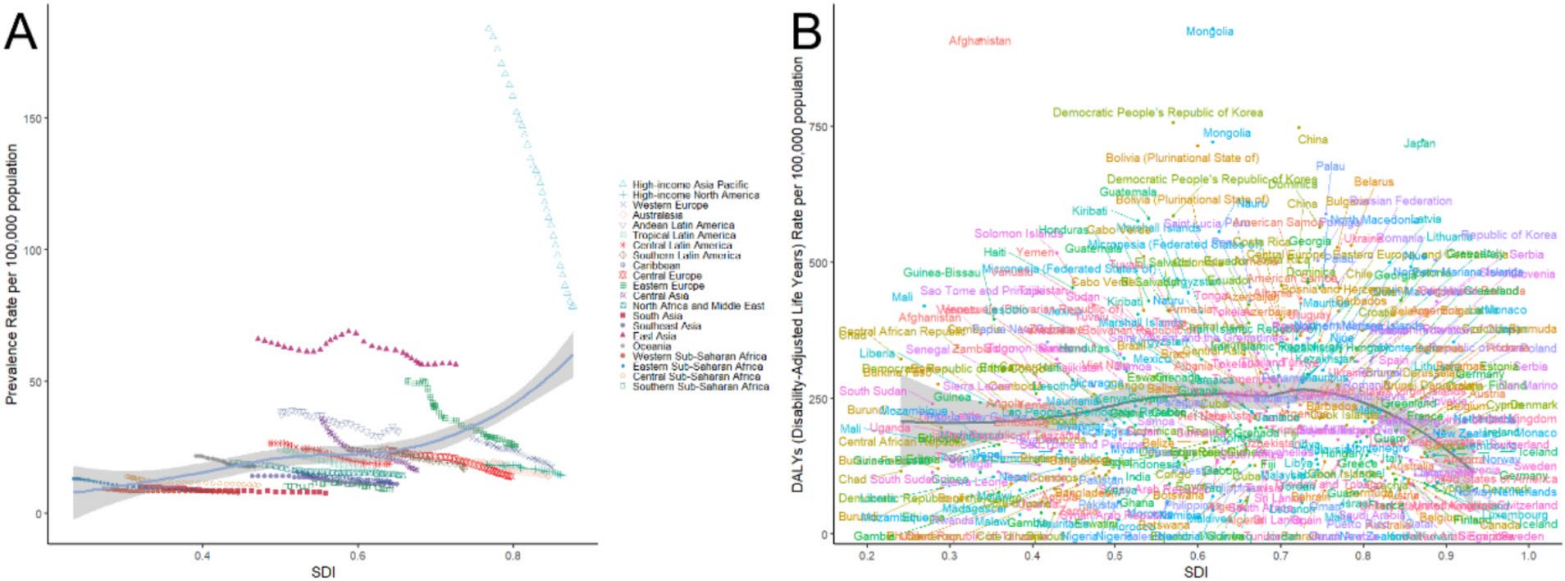
Relationship between gastric cancer prevalence and disability-adjusted life years (DALYs) rate with socio-demographic index (SDI), 1990–2021.

Figure 9 highlights the significant disparities in gastric cancer prevalence and disability-adjusted life year (DALY) rates across regions with varying levels of socio-demographic index (SDI). In Figure 9A, regions with high SDI levels demonstrated higher gastric cancer prevalence rates. The High-Income Asia Pacific region, including Japan and South Korea, had the highest prevalence, exceeding 150 cases per 100,000 population, making it the most affected area globally. High SDI regions such as High-Income North America and Western Europe had comparatively lower prevalence rates but still exceeded those of middle-and low-SDI regions. Middle-SDI regions, such as East Asia and Central Europe, exhibited moderate prevalence rates with relatively concentrated distributions. In contrast, low-SDI regions, such as sub-Saharan Africa, showed generally low prevalence rates with narrower distribution ranges. In Figure 9B, the DALY rates followed a similar trend to prevalence rates, with high-SDI regions, particularly the High-Income Asia Pacific region, displaying the highest DALY rates, significantly exceeding those of High-Income North America and Western Europe. Middle-SDI regions, including Eastern Europe and East Asia, showed broad distributions with moderate DALY levels. Low-SDI regions, such as sub-Saharan Africa, generally exhibited lower DALY rates, though certain countries still faced considerable disease burdens. These findings reveal marked differences in gastric cancer prevalence and DALY rates across regions with different SDI levels, reflecting the regional characteristics of disease burden globally. The results emphasize the need for region-specific strategies that consider local socioeconomic contexts to address the disparities in gastric cancer burden effectively.

### 3.8 Projections of gastric cancer burden by gender in China and globally over the next 15 years

**Figure 10:**
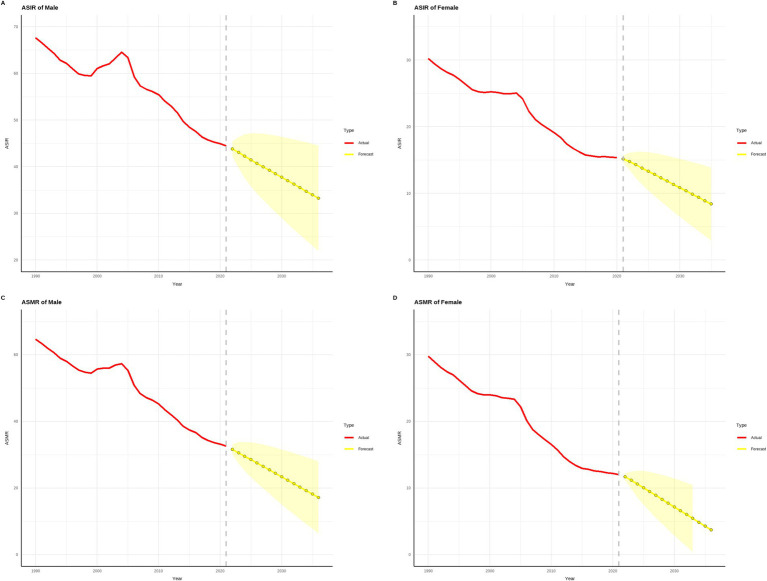
Projected trends in gastric cancer incidence and mortality rates in china over the next 15 years (2022–2036). (The red line represents the actual trend in gastric cancer incidence and mortality rates in China from 1990 to 2021, while the yellow dashed line and shaded area denote the projected trend and its 95% confidence interval for 2022–2036).

**Figure 11:**
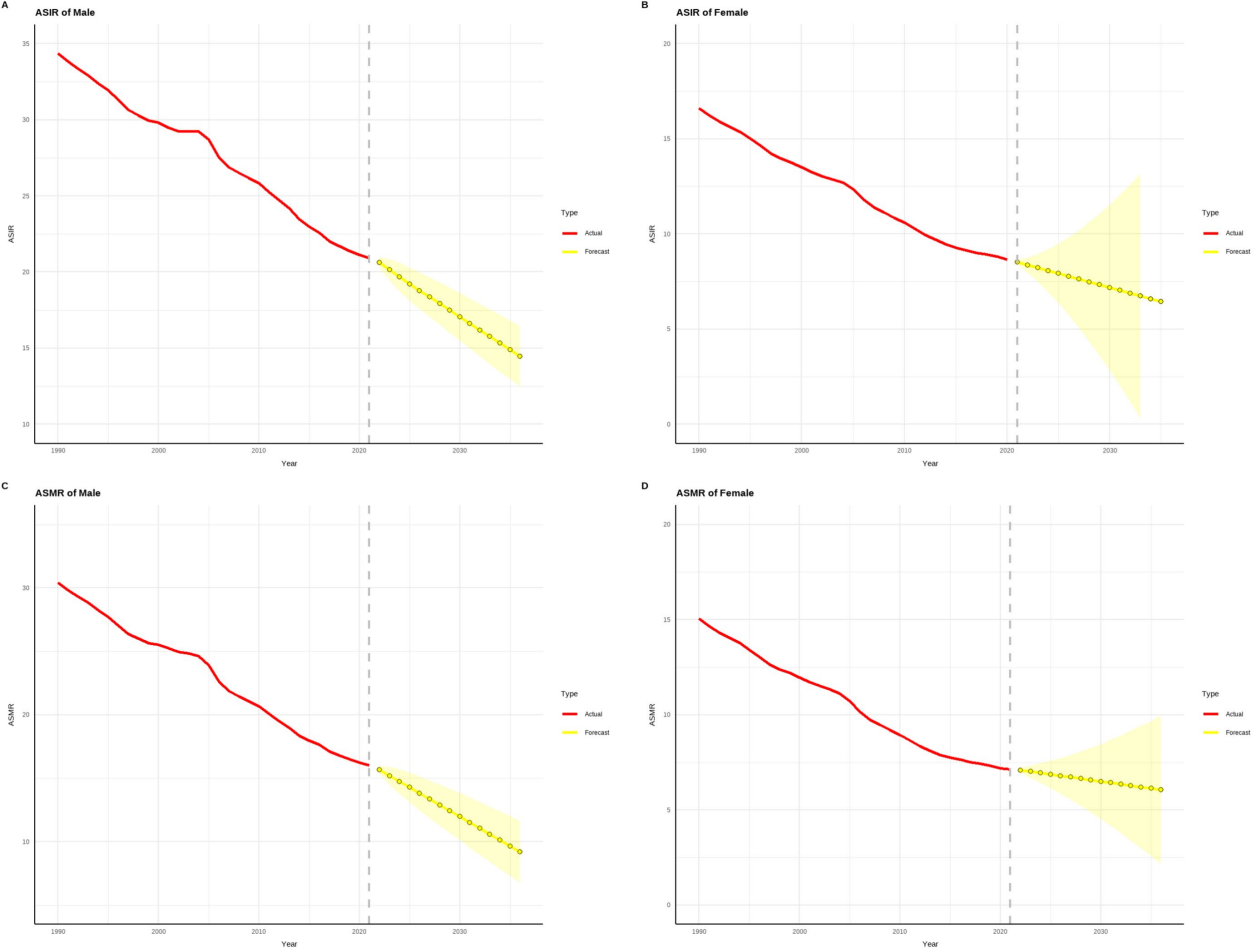
Projected trends in global gastric cancer incidence and mortality rates over the next 15 years (2022–2036). (The red line represents the actual trend in global gastric cancer incidence and mortality rates from 1990 to 2021, while the yellow dashed line and shaded area indicate the projected trend and its 95% confidence interval for 2022–2036).

Figures 10, 11 demonstrate that from 1990 to 2021, both the age-standardized incidence rate (ASIR) and age-standardized mortality rate (ASMR) of gastric cancer showed significant declines in China and globally. Projections for 2022 to 2036 suggest that this downward trend is likely to continue. In terms of ASIR, China’s rates for males decreased from approximately 70 per 100,000 in 1990 to around 40 per 100,000 in 2021, while females saw a reduction from around 35 per 100,000 to 20 per 100,000 over the same period. By 2036, these rates are projected to drop further, to about 25 per 100,000 for males and 12 per 100,000 for females. Globally, male ASIR declined from approximately 35 per 100,000 in 1990 to 20 per 100,000 in 2021, and female ASIR fell from around 20 per 100,000 to 10 per 100,000. By 2036, these are expected to decrease to about 12 per 100,000 for males and 6 per 100,000 for females. The ASMR followed a similar trend. In China, male ASMR decreased from around 60 per 100,000 in 1990 to 30 per 100,000 in 2021, and female ASMR dropped from approximately 30 per 100,000 to 15 per 100,000. By 2036, male and female ASMR are projected to decline further, to about 18 per 100,000 and 8 per 100,000, respectively. Globally, male ASMR fell from around 30 per 100,000 in 1990 to 15 per 100,000 in 2021, and female ASMR decreased from around 15 per 100,000 to 8 per 100,000. By 2036, these rates are expected to reach approximately 10 per 100,000 for males and 5 per 100,000 for females. Overall, the initial ASIR and ASMR values and their reduction magnitudes were higher in China compared to global figures, indicating significant progress in gastric cancer prevention and control in China, though baseline levels remain relatively high, necessitating continued efforts. Globally, males consistently exhibited higher ASIR and ASMR values than females, underscoring gender-related differences in gastric cancer burden. The projected data suggest that both China and the global population will continue to see reductions in gastric cancer burden over the next 15 years, highlighting the critical role of early screening, therapeutic interventions, and health improvement measures in sustaining this trend.

## 4 Discussion

Gastric cancer (12) remains a significant component of the global cancer burden, influenced by multiple factors. Epidemiological studies indicate that *Helicobacter pylori* infection is the primary risk factor for gastric cancer, accounting for over 60% of total cases (13–15). Additionally, unhealthy dietary habits (e.g., high-salt diets and pickled food intake), smoking, alcohol consumption, obesity, and genetic predisposition are critical contributors. The prevalence of *H. pylori* infection is significantly higher in low-and middle-income countries, reaching over 80% in sub-Saharan Africa compared to around 30% in Northern Europe and North America (16, 17). High-salt diets further exacerbate gastric cancer risk by damaging the gastric mucosal barrier and promoting inflammatory responses (17, 18).

Standard treatments for gastric cancer include surgical resection (e.g., gastrectomy), chemotherapy, radiotherapy, targeted therapy, and immunotherapy (19, 20). Five-year survival rates for early-stage gastric cancer exceed 90%, whereas those for advanced-stage cases remain below 20%. Notably, immunotherapies such as anti-PD-1 antibodies have shown promising results in treating advanced gastric cancer, offering new therapeutic options. Despite these advancements, the burden of gastric cancer remains substantial both in China and globally.

This study utilized long-term trend analysis from the GBD database to explore changes in gastric cancer burden. From 1990 to 2021, both China and the global population experienced significant reductions in age-standardized incidence rate (ASIR) and age-standardized mortality rate (ASMR), and projections indicate these trends will likely continue from 2022 to 2036. In China, ASIR decreased from 70 per 100,000 in 1990 to 40 per 100,000 in 2021 and is projected to reach 25 per 100,000 by 2036. ASMR dropped from 60 per 100,000 to 30 per 100,000 over the same period, with a further decline to 18 per 100,000 anticipated by 2036. Globally, ASIR fell from 35 per 100,000 in 1990 to 20 per 100,000 in 2021, and is expected to decline to 12 per 100,000 by 2036, while ASMR dropped from 30 per 100,000 to 15 per 100,000, with a projected decrease to 10 per 100,000 by 2036.

The GBD database and GLOBOCAN 2022 differ in methodology and objectives. The GBD database integrates multiple data sources and employs modeling to provide long-term trend analyses, making it suitable for observing temporal changes in disease burden. In contrast, GLOBOCAN emphasizes current annual cancer statistics, offering insights into the present burden. The two datasets reveal key differences: while the GBD database highlights a pronounced long-term decline in global gastric cancer burden, GLOBOCAN 2022 shows increasing cases in some low-income regions. These discrepancies likely arise from differences in data coverage and modeling assumptions. However, both datasets align in their overarching trends, indicating a gradual reduction in the global burden of gastric cancer.

This study revealed several important findings: significant declines in incidence and mortality: since 1990, global and Chinese gastric cancer ASIR and ASMR have shown marked declines, with China exhibiting the largest reductions. These improvements may be attributed to reduced *H. pylori* infection rates, heightened public health awareness, and enhanced medical resources. Large-scale *H. pylori* screening and treatment, early cancer detection programs, and promotion of healthy dietary habits in China have played vital roles in prevention. Persistent gender disparities: Males consistently exhibited higher ASIR and ASMR than females. From 1990 to 2021, Chinese males had ASIR rates approximately twice those of females, while globally, the male-to-female ratio was about 1.5. This disparity may result from higher rates of smoking, alcohol consumption, and unhealthy diets among males, as well as lower participation in early screening programs. Shifting peak age groups: the peak incidence age for gastric cancer has gradually shifted to older populations. In China, the male peak shifted from 55–59 years in 1990 to 65–69 years in 2021, with a similar trend observed among females. Globally, peak incidence also moved to older age groups. This shift likely reflects population aging and improved healthcare extending the survival of high-risk individuals (21). Regional disparities: developed regions generally experienced more significant declines in gastric cancer burden than developing regions. For instance, the High-Income Asia Pacific region (e.g., Japan and South Korea) had high baseline incidence rates but showed substantial reductions, whereas some low-income regions, such as sub-Saharan Africa, saw smaller declines or even increases in certain countries. These differences likely stem from unequal access to medical resources and varied implementation of prevention policies.

The strengths of this study lie in its large-scale, long-term trend analysis based on the GBD database, complemented by the latest GLOBOCAN 2022 data. By examining gastric cancer burden from temporal, spatial, gender, and regional perspectives, the study provides comprehensive insights and predictive models that inform future public health policy. Additionally, by comparing China and the global landscape, it identifies similarities and differences in prevention strategies, offering valuable references for high-burden countries.

However, this study has limitations. First, the reliance on modeled data may lead to inaccuracies, particularly for low-income countries where data completeness is limited. Second, predictions based on current trends may not account for future technological innovations or policy changes that could influence disease burden. Finally, methodological differences between the GBD and GLOBOCAN datasets may result in inconsistencies in specific indicators. Future research should integrate more real-world data and dynamically updated policy effects to enhance the reliability and scientific validity of analyses.

In summary, this study provides valuable scientific evidence on the changing trends of gastric cancer burden, offering crucial guidance for optimizing prevention strategies and public health policies. However, continued efforts to incorporate regional data and policy interventions are essential for refining projections and advancing global gastric cancer control.

## 5 Conclusion

The results underscore the importance of comprehensive prevention strategies focusing on early screening and *H. pylori* eradication, particularly in high-burden countries and regions. Optimizing medical resource allocation, promoting healthy dietary habits, and strengthening international collaboration are critical to further reducing the global gastric cancer burden. While this study provides robust data integration and trend analysis, incorporating regional data and policy effects will be crucial for refining future projections. Overall, the findings offer essential scientific support for optimizing gastric cancer prevention strategies and guiding public health policy and resource allocation.

## Data Availability

The original contributions presented in the study are included in the article/supplementary material, further inquiries can be directed to the corresponding author.

## References

[1] Bai X Zhu M He Y Wang T Tian D Shu J. The impacts of probiotics in eradication therapy of *Helicobacter pylori*. Arch Microbiol. (2022) 204:692. doi: 10.1007/s00203-022-03314-w, PMID: 36344628 PMC9640438

[2] Yang L Ying X Liu S Lyu G Xu Z Zhang X. Gastric cancer: epidemiology, risk factors and prevention strategies. Chin J Cancer Res. (2020) 32:695–704. doi: 10.21147/j.issn.1000-9604.2020.06.03, PMID: 33446993 PMC7797232

[3] López MJ Carbajal J Alfaro AL Saravia LG Zanabria D Araujo JM. Characteristics of gastric cancer around the world. Crit Rev Oncol Hematol. (2023) 181:103841. doi: 10.1016/j.critrevonc.2022.103841, PMID: 36240980

[4] Bray F Laversanne M Sung H Ferlay J Siegel RL Soerjomataram I. Global cancer statistics 2022: GLOBOCAN estimates of incidence and mortality worldwide for 36 cancers in 185 countries. CA Cancer J Clin. (2024) 74:229–63. doi: 10.3322/caac.21834, PMID: 38572751

[5] Niknam N Obanor S Lee LA. Endoscopic methods for the detection and treatment of gastric cancer. Curr Opin Gastroenterol. (2022) 38:436–42. doi: 10.1097/MOG.0000000000000867, PMID: 35881962

[6] Menon S. Serology-assisted endoscopic screening for gastric cancer. Gastrointest Endosc. (2024) 100:64–5. doi: 10.1016/j.gie.2024.02.036, PMID: 38879226

[7] Murray CJL. Findings from the global burden of disease study 2021. Lancet. (2024) 403:2259–62. doi: 10.1016/S0140-6736(24)00769-4, PMID: 38762327

[8] Lou HR Wang X Gao Y Zeng Q. Comparison of ARIMA model, DNN model and LSTM model in predicting disease burden of occupational pneumoconiosis in Tianjin, China. BMC Public Health. (2022) 22:2167. doi: 10.1186/s12889-022-14642-3, PMID: 36434563 PMC9694549

[9] Chen H-S Zeichner S Anderson RN Espey DK Kim H-J Feuer EJ. The joinpoint-jump and joinpoint-comparability ratio model for trend analysis with applications to coding changes in health statistics. J Off Stat. (2020) 36:49–62. doi: 10.2478/jos-2020-0003, PMID: 32713989 PMC7380682

[10] Zhang Y Liu J Han X Jiang H Zhang L Hu J. Long-term trends in the burden of inflammatory bowel disease in China over three decades: a joinpoint regression and age-period-cohort analysis based on GBD 2019. Front Public Health. (2022) 10:994619. doi: 10.3389/fpubh.2022.994619, PMID: 36159285 PMC9490087

[11] Zhu B Wang Y Zhou W Jin S Shen Z Zhang H. Trend dynamics of gout prevalence among the Chinese population, 1990-2019: a joinpoint and age-period-cohort analysis. Front Public Health. (2022) 10:1008598. doi: 10.3389/fpubh.2022.1008598, PMID: 36311630 PMC9602928

[12] Pakbin B Allahyari S Dibazar SP Zolghadr L Chermahini NK Brück WM. Effects of probiotic *Saccharomyces boulardii* supernatant on viability, nano-mechanical properties of cytoplasmic membrane and pro-inflammatory gene expression in human gastric cancer AGS cells. Int J Mol Sci. (2023) 24:7945. doi: 10.3390/ijms24097945, PMID: 37175663 PMC10178855

[13] Pan K-F Li W-Q Zhang L Liu W-D Ma J-L Zhang Y. Gastric cancer prevention by community eradication of *Helicobacter pylori*: a cluster-randomized controlled trial. Nat Med. (2024) 30:3250–60. doi: 10.1038/s41591-024-03153-w, PMID: 39079993

[14] Chen Y-C Malfertheiner P Yu H-T Kuo C-L Chang Y-Y Meng F-T. Global prevalence of *Helicobacter pylori* infection and incidence of gastric cancer between 1980 and 2022. Gastroenterology. (2024) 166:605–19. doi: 10.1053/j.gastro.2023.12.022, PMID: 38176660

[15] Shirani M Shariati S Bazdar M Sojoudi Ghamnak F Moradi M Shams Khozani R. The immunopathogenesis of *Helicobacter pylori*-induced gastric cancer: a narrative review. Front Microbiol. (2024) 15:1395403. doi: 10.3389/fmicb.2024.1395403, PMID: 39035439 PMC11258019

[16] McMahon MV Taylor CS Ward ZJ Alarid-Escudero F Camargo MC Laszkowska M. *Helicobacter pylori* infection in the United States beyond NHANES: a scoping review of seroprevalence estimates by racial and ethnic groups. Lancet Reg Health Am. (2024) 41:100890. doi: 10.1016/j.lana.2024.100890, PMID: 40321653 PMC12049726

[17] Sadeghi A Nouri F Taherifard E Shahlaee MA Dehdari EN. Estimates of global and regional prevalence of *Helicobacter pylori* infection among individuals with obesity: a systematic review and meta-analysis. Infection. (2024) 52:1223–34. doi: 10.1007/s15010-024-02244-7, PMID: 38594573

[18] Kronsteiner-Gicevic S Thompson AS Gaggl M Bell W Cassidy A Kühn T. Adding salt to food at table as an indicator of gastric cancer risk among adults: a prospective study. Gastric Cancer. (2024):714–21. doi: 10.1007/s10120-024-01502-9 38630317 PMC11193689

[19] Sharma S Carey N McConnell D Lowery M O’Sullivan J McCullagh L. Systematic review of economic evaluations of systemic treatments for advanced and metastatic gastric cancer. PharmacoEconomics. (2024) 42:1091–110. doi: 10.1007/s40273-024-01413-8, PMID: 39060831 PMC11405472

[20] Triantafillidis JK Konstadoulakis MM Papalois AE. Immunotherapy of gastric cancer: present status and future perspectives. World J Gastroenterol. (2024) 30:779–93. doi: 10.3748/wjg.v30.i8.779, PMID: 38516237 PMC10950642

[21] Pakbin B Allahyari S Dibazar SP Peymani A Haghverdi MK Taherkhani K. Anticancer properties of *Saccharomyces boulardii* metabolite against colon cancer cells. Probiotics Antimicrob Proteins. (2024) 16:224–32. doi: 10.1007/s12602-022-10030-w, PMID: 36547769

